# Ex Vivo Models Simulating the Bone Marrow Environment and Predicting Response to Therapy in Multiple Myeloma

**DOI:** 10.3390/cancers12082006

**Published:** 2020-07-22

**Authors:** Konstantinos Papadimitriou, Ioannis V. Kostopoulos, Anastasia Tsopanidou, Nikolaos Orologas-Stavrou, Efstathios Kastritis, Ourania E. Tsitsilonis, Meletios A. Dimopoulos, Evangelos Terpos

**Affiliations:** 1 Department of Biology, School of Sciences, National and Kapodistrian University of Athens, 15784 Athens, Greece; konstantinos.pap@hotmail.com (K.P.); anastasia_ts@hotmail.com (A.T.); norologas@biol.uoa.gr (N.O.-S.); rtsitsil@biol.uoa.gr (O.E.T.); 2 Department of Clinical Therapeutics, School of Medicine, National and Kapodistrian University of Athens, 11528 Athens, Greece; ekastritis@med.uoa.gr (E.K.); mdimop@med.uoa.gr (M.A.D.)

**Keywords:** multiple myeloma, 3D model, bone marrow microenvironment, personalized therapy, precision medicine

## Abstract

Multiple myeloma (MM) remains incurable despite the abundance of novel drugs. As it has been previously shown, preclinical 2D models fail to predict disease progression due to their inability to simulate the microenvironment of the bone marrow. In this review, we focus on 3D models and present all currently available ex vivo MM models that fulfil certain criteria, such as development of complex 3D environments using patients’ cells and ability to test different drugs in order to assess personalized MM treatment efficacy of various regimens and combinations. We selected models representing the top-notch ex vivo platforms and evaluated them in terms of cost, time-span, and feasibility of the method. Finally, we propose where such a model can be more informative in a patient’s treatment timeline. Overall, advanced 3D preclinical models are very promising as they may eventually offer the opportunity to precisely select the optimal personalized treatment for each MM patient.

## 1. Introduction

Multiple myeloma (MM) is a haematological malignancy of the plasma cells (PCs), characterized by the accumulation of clonal plasma cells in the bone marrow (BM) and overproduction of monoclonal immunoglobulins (Igs) [1,2]. Interaction of aberrant PCs with osteoclasts, osteoblasts, and several immune subsets in the BM leads to manifestation of MM symptoms, including anaemia, bone fractures, susceptibility to infections, hypercalcemia, fatigue, and pain, whereas accumulation of Igs might lead to consequences such as renal failure [1,2,3,4]. The BM microenvironment is the primary modulator of both malignant transformation and disease progression, where myeloma cells adhere, proliferate, and migrate [5,6,7]. If MM undergoes clonal evolution and drug resistance, it may progress to plasma cell leukemia, an aggressive BM-independent disease, where aberrant PCs spread into circulation or additionally develop extramedullary plasmatocytoma, where MM cells spread in soft-tissues with adverse clinical outcome [8]. To date, and despite the abundance of therapeutic choices, MM remains an incurable cancer, usually with an overall survival (OS) of less than 10 years [9,10].

As for treatment, an impressive progress has been made during the past 20 years, gradually rendering MM from a disease with limited OS to a chronic disease with alternating symptomatic and remission phases. Moving beyond alkylating agents and autologous stem cell transplantation (ASCT), the introduction of immunomodulatory drugs (IMiDs), proteasome inhibitors, histone deacetylase (HDAC) inhibitors, and monoclonal antibodies tremendously shifted the therapeutic approach of MM [11,12,13,14,15]. The plethora of novel agents and therapeutic approaches necessitates precise and effective methodologies for the rapid assessment of the efficacy of these agents for each individual patient separately. Since the traditional preclinical 2D models have failed to predict disease progression due to their inability to simulate the BM microenvironment, 3D platforms are exploited as they likely mimic ex vivo the in vivo cellular and non-cellular interactions.

## 2. Study Aim and Design

The aim of this review is to present 3D ex vivo platforms specifically developed for MM and to discuss the advantages and limitations of each model for assessing the personalized MM treatment efficacy of various regimens and combinations. After an extensive bibliographical search under the terms “multiple myeloma” and “3D culture” or “ex vivo assay” or “3D platform” or “preclinical model”, we selected publications that fulfilled the following criteria: (a) refer to systems that emulate the BM microenvironment, i.e., they use both MM cells and other cell types and/or substances known to participate and regulate cell interactions in the BM microenvironment; (b) apply to MM cells developed in 3D structures; (c) provide results on at least one drug tested; (d) the outcome is evaluated on the basis of MM cell viability following exposure to the drug(s); and (e) the platforms are developed at a personalized patient-specific level. We selected 14 models that, in our opinion, represent the top-notch full range of MM platforms developed to date, classified according to the type of material and methodology used to simulate the BM microenvironment. We also briefly refer to some promising approaches partially fulfilling the aforementioned criteria set. We further explain why 3D cultures are the current model of choice in studying the BM microenvironment in MM.

## 3. Role of the BM Microenvironment in MM

The pathophysiology of MM consists of both the primary oncogenic events occurring in myeloma cells (i.e., chromosomal translocations, structural abnormalities, gene mutations), as well as of extracellular factors and dynamic interactions between clonal cells and the BM components. Since MM cells grow and proliferate almost exclusively within the BM, it is rational to consider that the BM microenvironment plays the most critical role in the development and expansion of MM cells [16]. The BM microenvironment is subdivided into two major compartments: the cellular and the non-cellular. The former consists of all hematopoietic cells of erythroid, myeloid, and lymphoid lineages as well as non-hematopoietic cells including BM stromal cells (BMSCs), fibroblasts, osteoblasts, osteoclasts, endothelial cells, adipocytes, and cells of the blood vessels. The non-cellular compartment comprises the extracellular matrix (ECM; consisting of collagen, glycoproteins, matrix proteoglycans, and glycosaminoglycans) and the liquid milieu (cytokines, growth factors, and chemokines), which are produced and/or altered by the cellular compartment. The two BM microenvironment compartments interact dynamically with malignant cells and likely work synergistically to support MM cell survival and progression, as shown both in vitro and in vivo [17,18,19].

Although MM cells have been reported to interact with almost every BM bystander cell type, BMSCs have the most direct effect on MM cell growth, proliferation, and drug resistance via both cytokine secretion and cell-to-cell adhesion [20,21]. BMSCs primarily secrete interleukin (IL)-6, an important growth and survival factor, which, in turn boosts the secretion of vascular endothelial growth factor (VEGF), the key angiogenic factor [22,23]. Additionally, BMSCs produce insulin-like growth factor (IGF)-1 and IL-8 that further induce MM cell growth, migration, MM-associated angiogenesis, and osteolysis through distinct molecular pathways [24]. Contact of MM cells with BMSCs and/or ECM components may trigger cell adhesion-mediated drug-resistance mechanisms, through which MM cells escape the cytotoxic effects of anticancer therapies (e.g., of doxorubicin, melphalan, vincristine, dexamethasone, and mitoxantrone) [25,26]. Interactions between MM cells and BMSCs are also mediated by Notch, which confers resistance to drugs via increased secretion of IL-6, IGF-1, and VEGF [17]. Finally, BMSCs and MM cells also interact through a complex intracellular network of miRNA-bearing exosomes which regulate MM cell gene expression [16].

The BM microenvironment has been the target of many novel agents aiming at the disruption of the mechanisms that support MM cell growth and disease progression. We will focus mostly on the role of BMSCs, as these cells are the most widely used in MM 3D co-cultures to reproduce ex vivo the protective/supportive effect of the BM milieu on MM cells against various therapeutic regimens.

## 4. The Need for Personalized Preclinical BM Models

The therapeutic choice in MM is based on clinical factors such as patients’ age, prognostic parameters, and comorbidities. However, MM is highly heterogeneous in terms of phenotype, clinical symptoms, and particularly genomic aberrations, and current in vitro molecular modelling may only partially predict therapeutic efficacy [27,28,29,30,31]. Therefore, the development of a rapid and effective methodology for evaluating the efficacy of several therapeutic agents based on the patient-individualized profile would be of utmost significance [32,33,34,35,36]. In the same context, and beyond finding a specific sensitive target, the application of a suitable preclinical model would prevent ineffective therapy of resistant MM cells and unwanted toxicities [37,38,39].

Clinical studies utilizing novel agents have shown that some molecular subgroups of patients may benefit from administration of specific drugs [40,41,42]. Although molecular testing might highlight the molecular sensitivity of myeloma cells, the BM microenvironment also plays a key role in disease progression and resistance to therapy [43]. Thus, to better predict which drug or therapeutic scheme will benefit each individual patient, a successful preclinical model should incorporate both the molecular profiling of MM cells and the BM microenvironment as a whole. The outcome of such a platform would form a personalized therapy profile that could ideally apply in daily clinical practice affecting clinical decision-making.

## 5. Personalized Clinical Assays for MM Patients: From 2D to 3D BM Cell Cultures

A variety of different strategies has been proposed, aiming to create an ex vivo platform that will simulate the BM microenvironment and/or test treatment efficacy of various MM agents. In the clinical setting, such an ex vivo platform should bear certain characteristics that will permit its eventual use for personalized drug-screening in patients undergoing routine treatment. Given the narrow time-frame for subsequent decision-making, an ideal drug-testing platform should be: (1) time-efficient, (2) easy-to-perform, (3) highly reproducible, (4) able to simultaneously process many samples, and (5) cost-effective.

2D monocultures that use established MM cell lines have been thoroughly tested in the past. Even though this type of culture is suitable for high-throughput drug screening, it cannot reproduce the complexity of the BM microenvironment and does not reflect the heterogeneity of MM patients’ cells, generating models that are more generic and less (if at all) personalized [44]. The use of primary patient-derived MM cells in a 2D monoculture maintains the heterogeneity of the sample; however, the microenvironment is still missing in this BM simulation. Moreover, when in culture, primary MM cells may gradually change morphology and phenotype, possibly de-differentiating into immature resilient phenotypes [45]. The next step in the evolution of 2D cultures was the generation of a co-culture system in which primary MM cells were cultured together with autologous BMSCs, mesenchymal stem cells (MSCs), osteoclasts or immature dendritic cells. Depending on the accompanying cell type, primary MM cells could stabilize or even increase their numbers and, in some cases, develop an enhanced drug resistance [46,47]. Nevertheless, cells are still mostly grown on a 2D layer interacting in a spatially limited manner.

3D culture systems have shown their superiority in simulating more realistically the in vivo cellular environment, as they quite accurately mimic normal cell morphology, proliferation, differentiation, and migration. This is due to the fact that the cell cytoskeleton is expanded in all directions, while the cell-to-cell and cell-to-surface interactions that occur in a 3D space are very similar to the interactions observed in vivo [48,49]. Moreover, the deposition of ECM in 3D cultures promotes cells to form 3D spheroids and plays an important role in cancer progression and invasion [50]. With respect to MM, it has been proven that cytokine production (e.g., IL-11, HGF, and IL-6) is higher in 3D *versus* 2D cultures [51]. Furthermore, 3D models also seem to better recapitulate the effect of anti-myeloma drugs (e.g., melphalan and bortezomib), thus highlighting the existence of drug-resistant MM clonal compartments [52].

## 6. 3D Ex Vivo Platforms Using Gel Scaffolds

The first effort to build a 3D model for MM was reported in 2008 by Kirshner et al. [52] (Table 1). In this pioneering model termed “rEnd-rBM”, plates were pre-treated with fibronectin/collagen type I, creating the reconstructed endosteum-marrow junction (rEnd) compartment. Patient BM mononuclear cells (BMMCs) were subsequently added in a jellified scaffold of Matrigel/fibronectin, thus creating the recombinant BM (rBM) compartment, and were further cultured in growth medium supplemented with the patient’s blood plasma. After treatment with melphalan or bortezomib, cells were removed from the scaffold to assess apoptosis and reduction in the number of clonal cells. The model was validated by examining the rBM environment with confocal microscopy and immunohistochemistry, showing that the rBM resembled the natural BM niche as it maintained the architecture of the human BM and also supported the expansion of the MM clone. Most importantly, this was the first 3D model to study the effect of agents separately on the different cellular compartments of stromal cells, hematopoietic cells, and myeloma cells, thus providing the ability to investigate the precise cell target(s) of each agent [52].

Some years later, Parikh et al. [53] and then Huang et al. [54] used an optimized protocol of Kirshner’s rBM model, in order to study the activation of the STAT3 pathway in 3D versus conventional 2D cultures. Specifically, the 3D culture was formed in a well with histogel that could be easily fixed, thus allowing for further histologic processing and immunocytochemical studies. Huang et al. [54] showed that the STAT3 pathway was activated when cells were cultured in 3D, while it remained inactive in conventional 2D cultures. Inhibition of the STAT3 pathway using the pharmacological agent Stattic significantly decreased the viability of MM cells and increased their susceptibility to bortezomib. These results were important as MM cells within the BM are also STAT3 active, thus implying that 3D models are superimposing 2D approaches and can better mimic the in vivo state [54].

De la Puente et al. [55] developed a patient-derived model, termed “3D tissue-engineered BM culture model” (3DTEBM), consisting of BM supernatant derived from MM patients and autologous BM cells, combined in a gel scaffold prepared from each patient’s plasma fibrinogen. In particular, the 3D matrices were developed via cross-linking of fibrinogen at various CaCl_2_ concentrations in the presence of tranexamic acid, in order to formulate a sustaining gel in 96-well plates. 3DTEBM allowed for the detection of drug uptake by MM cells throughout the 3DTEBM depth and was also used to study the effect of hypoxia on the development of drug resistance to various antimyeloma drugs such as carfilzomib and bortezomib. This model could portray the in vivo drug concentration gradient for a more representative ex vivo drug-effect evaluation, and also proved to support MM cell proliferation more efficiently than standard 2D and 3D cultures [55,56]. However, despite these promising results, the utilization of the 3DTEBM in the frame of a clinical trial has not been reported to date [56].

A report on gel scaffolds using exclusively PuraMatrix hydrogel (BD Biosciences, Franklin Lakes, NJ, USA) was published by Jakubicova et al. [57]. In this approach, BM mononuclear cells were obtained from BM aspiration by red blood cell lysis, and the adherent fraction containing MSCs was expanded for several days, while the soluble fraction containing the MM cells was frozen. MSCs were seeded on the surface of the gel, and MM cells were added after culture establishment. To assess viability, carboxyfluorescein succinimidyl ester (CFSE)-labelled MM cells were stained with Annexin V/7-aminoactinomycin D (7-AAD) to distinguish apoptotic and necrotic cells. Eight therapeutic agents were tested in total, including the IMiDs pomalidomide, lenalidomide, thalidomide. The authors showed a different drug-assessed profile for each patient tested, noting that patients’ cells were more resistant to IMiDs in 3D versus 2D cultures. MSCs were confirmed to keep their MSC phenotype as shown by flow cytometry. Analysis of cytokine secretion and ECM molecules revealed high similarity with the in vivo MM microenvironment composition, while active osteogenesis and osteoblastic differentiation were also observed. Subsequently, gene expression analysis underlined the differences between 3D versus 2D models and highlighted the importance of the activation of osteogenesis.

In another approach, 3D tumor spheroid cultures were formed on alginate bead culture by suspending MM cells in a medium-viscosity sodium alginate dissolved in saline, which polymerised when CaCl_2_ was added. Based on this model, Arhoma et al. [58] reported the effects of Tumour Necrosis Factor-Related Apoptosis-Inducing Ligand (TRAIL) and HDAC inhibitors in six human MM cell lines and primary plasma cell leukemia. Using caspase-3 activity assay to confirm apoptotic responses to the agents and CellTitler-Glo viability testing, the researchers showed that combination therapy with TRAIL and HDAC inhibitors induced apoptosis, and MM cells in 3D spheroid cultures were more sensitive to drug treatment compared to suspension cell culture.

Based on computational modelling, Ji et al. [59] introduced the hybrid multi-scale agent-based model (HABM), which combines two mathematical systems, ordinary differentiated equations (ODEs) and an agent-based model (ABM). The purpose of the study was to predict the effect of bortezomib and the IMiDs lenalidomide and thalidomide in an environment that simulates MM cell growth within the BM, taking into consideration the important role of immune cells in this process. In order to validate their mathematical model, the team developed collagen-based 3D co-cultures of patients’ MM cells and MSCs, and proved that the proposed model was of high accuracy. Although such models have not been utilized in daily clinical practice, a computational approach that takes into account the dynamic interplay between MM cells, immune cells, and alterations in the BM microenvironment during disease progression would be of great importance for the development of a one-step therapy prediction model at no cost.

In an effort to exploit the advances of cellular immunotherapy against MM, Braham et al. [60] recently investigated the use of a novel class of engineered immune cells, called TEGs (i.e., αβT cells engineered to express a defined tumor-specific γδTCR), in targeting and eliminating primary MM cells. Researchers used endothelial progenitor cells and MSCs to form a network inside the Matrigel matrix, thus creating a 3D model that could facilitate the survival of primary MM cells. They then added TEGs that migrated inside the 3D structure finding their targets. The 3D model was highly superior when compared to a similar 2D approach and considered appropriate for clinical testing as it was successfully applied to 13 patients’ samples. The same 3D model was also implemented to test liposomal delivery of drugs, such as doxorubicin and bortezomib [61]. This study showed increased cytotoxic effects of loaded liposomes against MM cells compared with free drugs, using either an MM cell line or primary BM-derived cells.

In our opinion, the most elegant work on gel scaffolds has been reported by Silva et al. [62], who published the ex vivo mathematical myeloma advisor (EMMA), a tool for predicting the 3-month clinical response within five days by using a digital image analysis algorithm, mathematical models, and pharmacokinetic data. The model was tested using 31 drugs or combinations on BM samples from 52 patients, where primary MM cells were seeded in collagen type I and pre-established human-derived stroma. After the addition of the drug with a robotic pipettor, the plates were placed in a motorized microscope linked to an incubator, and cultures underwent complete imaging every 3 min for four consecutive days. Viability was calculated by a digital algorithm, which detected live and dead cells from the presence or absence of membrane motion over time. The simulation of different clinical treatments was performed by using the mathematical model of chemosensitivity and the pharmacokinetic properties of the selected therapeutic regimen. Impressively, EMMA was able to predict clinical responses to single agents and regimens in 41 out of 52 patients (79%). It also demonstrated that 66% of MM patients have received at least one ineffective drug, while 33% of the patients could have been treated with more potent drugs.

## 7. 3D Ex Vivo Platforms Using Solid Scaffolds

The BM is a mineralized environment with structural stiffness and complicated geometry with spatial dimensionality. These characteristics, in conjunction with neighbouring cells and secreted soluble factors, regulate cellular behaviour [69]. Based on this, Reagan et al. [63] attempted to recreate the BM microenvironment by stimulating MSCs to undergo osteogenic differentiation inside porous silk scaffolds. To achieve this, researchers developed tissue-engineered bone (TE-bone) comprising cylinder-shaped aqueous silk fibroin scaffolds with pores of 500–600 μm in diameter. MSCs were seeded and cultured with osteogenic media and shortly after, transduced MM cell lines or Cell-Tracker stained patient-derived MM cells were added. Viability after bortezomib treatment was measured using bioluminescence imaging (BLI) and was imaged with confocal microscopy, showing drug resistance in 3D compared to 2D cultures. Notably, researchers also showed alterations in MSC miRNAs and identified overexpression of miR-199 that enhanced the osteogenic potential of normal BM cells. On the contrary, downregulation of miR-199 in MM caused increased tumor cell accumulation.

## 8. 3D Ex Vivo Platforms Using Bioreactors

Bioreactors are devices that are able to support a biologically active environment by keeping cells in a reaction vessel, while, in continuous bioreactors, there is a perpetual input of new medium and output of cellular mass. The advantage of bioreactors for cell cultures is the generation of a constant dynamic fluid flow in the culture volume that ensures a continuous balance between culture mass and the renewal of culture medium. Currently, the only bioreactor that has been used in the setting of MM is the RSS Bioreactor (RCCS^TM^, Synthecon Inc., Houston, TX, USA).

In their recent publication, Belloni et al. [64] used patients’ myeloma tissue explants, normal skin, and bone, in order to preserve the tissue’s architecture inside the bioreactor vessels. MM specimens from vertebroplasty were cultured in the bioreactor with bortezomib, melphalan, dexamethasone, and the anti-VLA-4 monoclonal antibody natalizumab to evaluate viability and cytotoxicity induced by each drug. This model allowed for a long-term maintenance and proliferation of resistant MM clones, while FISH analysis indicated that a 13q14.3 clone preferentially populated the bioreactor. Bonomi et al. [65] used the RCCS bioreactor in another interesting setting; they loaded MSCs with paclitaxel (PTX) and used them as “trojan horses” to deliver the drug in situ. Initially, MSCs were primed with PTX and then transferred to a 3D dynamic culture of the RPMI 8226 cell line. After 72 h of co-culture, cells were harvested and embedded for morphological analysis to assess cellular necrosis. The inhibitory activity of PTX-primed MSCs was comparable to that of PTX alone, showing that the loaded-MSC strategy could be utilised to deliver drugs into the BM.

## 9. 3D Cultures Using Microfluidics

Microfluidics can be loosely defined as the controllable manipulation of fluids that are geometrically constrained at a small scale in order to control the cellular environment [70]. Khin et al. [66] described a microfluidic dose-response platform for the in vitro screening of drugs, accompanied by a computational model of clinical response. The 3D BM microenvironment consisted of patients’ MSCs and MM cells, ECM, and growth factors cultured in a collagen type I/culture media mix, which was developed inside the 3D cell-culture μ-slide (Ibidi, Gräfelfing, Germany). Each slide contained three chambers that formed a linear chemical gradient via passive diffusion, and slides were placed under a microscope inside a culture chamber. The model was used to test the effect of bortezomib and melphalan, and cell viability was assessed through detection of membrane motion with image analysis. The results of drug testing in four patients’ samples were used to parameterize computational models, which by combining the in vitro results together with genomic information could make predictions of the clinical response.

## 10. 3D Cultures Using Animal Models

Animal xenograft models are very important tools in our understanding of the pathogenesis of MM and the mode of action of novel agents [71]. Searching for relevant animal models that fulfil all set criteria, we selected only two publications, mainly due to the fact that the majority of reported models were not developed on the context of a personalized patient approach.

Martowicz et al. [67] used chicken embryos, as these are suitable for xenotransplantation due to a lack of adaptive immune responses until hatching [72]. The model combined the advantage of a 3D culture system with ex vivo development of chicken embryos. In this approach, green fluorescence protein (GFP)-transfected MM cell lines were cultured in spheroids along with MSCs using a collagen matrix. The spheroids were treated with bortezomib and subsequently were grafted on the chorioallantoic membrane of chicken embryos, where tumor growth was monitored. According to the authors, this model provides an inexpensive tool that, besides drug screening, can also be useful for the analysis of myeloma-induced angiogenesis.

Aiming to create the most realistic human BM milieu, Calimeri et al. [68] generated a severe combined immunodeficiency (SCID)-synth-hu mouse model. This was the first system for the in vivo expansion of human MM cells on a synthetic platform. For this purpose, human BMSCs isolated from MM patients were injected in vitro into a 3D bone-like poly-ε-caprolactone polymeric scaffold (PCLS) and surgically implanted subcutaneously in SCID mice. After three weeks, patients’ MM cells were injected into the implanted PCLSs and their growth was monitored for the next 3–4 weeks by detecting human heavy or light chains in mouse serum. Alternatively, patients’ BM mononuclear cells containing both BMSCs and MM cells were injected into PCLSs which were then implanted into SCID mice. Treatment with bortezomib and dexamethasone resulted in inhibition of MM cell growth in vivo as demonstrated by the decrease of paraprotein levels in mouse sera together with the detection of apoptotic MM cells in retrieved PCLSs. Overall, the results of these experiments were encouraging and revealed that this model is suitable for large-scale in vivo preclinical screening for novel anti-MM drugs.

## 11. 3D Models Partially Fulfilling the Criteria Set

There are also some models worth mentioning, even though they do not fulfil all criteria we initially set. Fairfield et al. [73] developed the first 3D model based on adipose tissue (BMAT model). In particular, this model consisted of MSCs on silk scaffolds, cultured in adipogenic media driving differentiation of adipocytes. Overall, this approach set the basis for the study of the intricate interactions between BMAT and tumour cells even if no patient-derived cells were used. In another interesting approach, Cetin et al. [74] introduced an innovative functional assay to evaluate ex vivo drug sensitivity of single MM cells, based on measuring their mass accumulation rate with a microfluidic device. The model provided a quick and reliable prediction of therapeutic responses in clinical samples; nevertheless, the model referred only to MM cells, without including the BM microenvironment in its ex vivo simulation. Recently, an alternative 3D simulation approach was reported by Delgado-Calle et al. [75] to examine the effects of the novel therapeutic agent Aplidin. Using biopsy punches, 3.5 mm mouse bone disks were cut and placed in plates, where 5TGM1 MM cells were added. The study explored the mechanism of action of Aplidin, showing that it has anti-myeloma activity per se, and that it enhances the activity of proteasome inhibitors to hinder MM cell growth and bone destruction.

## 12. Classification and Evaluation of the 3D MM Models

All these models may have particular pros and cons with respect to each other. To give a better overview of these approaches, Table 1 summarizes the most important 3D simulation models based on three major parameters: duration of experiment, cost, and feasibility of the methodology used.

Τime-to-treatment decision is critical in the clinical setting of MM; hence, an ideal 3D simulation model should deliver results as soon as possible. High-throughput drug screening is also desirable: if many drugs are tested on the same platform at a given time, the results provided are more clinically relevant. Under this context, the SCID animal model [68] that requires a minimum of six weeks, or the rEND model [52] that requires more than 30 days are probably too long. On the contrary, the gel scaffold model of Silva et al. [62] seems advantageous as it allows the parallel testing of many drugs (up to 1536 in multi-well plates on a single run), provides automated image analysis of every well with no need of staining, and, most importantly, the results can be available in less than five days.

The total cost of a preclinical model is also an important aspect, especially when intended for large-scale screening processes. When comparing the different scaffold categories, it is evident that gel scaffolds combine high-throughput screening with low-cost materials and instrumentation. Moreover, costs can be kept low when cell viability or apoptosis is assessed by methods that do not require complex instrumentation (i.e., flow cytometry, confocal microscopy, computer-based image analysis). In this context, the methods of Arhoma et al. [58], Ji et al. [59], and Silva et al. [62] are the most cost-effective. On the contrary, bioreactors require sophisticated devices and are costly due to expensive consumables and maintenance. Concerning animal models, the need of one SCID mouse for every drug tested translates into approximately a dozen of mice euthanized for every patient, thus increasing the cost, but also raising ethical issues; on the other hand, the egg model might reduce cost, but the implementation of the methodology described by Martowicz et al. [67] for many patients and drugs requires specifically organized facilities.

As for feasibility, the benefits of easy-to-handle materials and method simplicity favor gel scaffolds. Specifically, the approach of Jakubikova et al. [57] seems to be the most applicable, as it uses Puramatrix, a simple commercially available material, and assesses apoptosis by a commonly used flow cytometric assay.

In MM patients, blood plasma profiling for markers beyond monoclonal M-protein and free light chain ratio include, among others, VEGF, transforming growth factor (TGF), epidermal growth factor (EGF)-2, and monocyte chemotactic protein-3, which in principle aim at patient stratification into distinct prognostic groups. However, evaluation of the efficacy of MM models using surrogate markers has been implemented in a very limited number of studies. Puente et al. [55] performed the most thorough cytokine profiling in 3D culture supernatants (38 cytokines including VEGF, TGF, and EGF), supporting the utility of 3D versus 2D cultures. Ferrarini et al. [76] showed differences in VEGF levels and matrix metalloproteinase (MMP)-2/MMP-9 activity in 3D cultures exposed or not to bortezomib. Therefore, selected surrogate markers, likely including cytokines, chemokines, growth factors, and possibly also extracellular vesicles secreted by MM cells in the 3D culture milieu, could assist the evaluation of the efficacy of 3D models and facilitate their utility for drug screening.

## 13. Time-Frame for the Application of an Ex Vivo 3D Platform in MM Patient Treatment

According to the most recent European Society of Medical Oncology guidelines for MM diagnosis, treatment, and follow-up [77], patients that have been diagnosed with MM and are eligible for ASCT undergo the following standard treatment: induction therapy consisting of 3-drug regimens (which almost always contain a proteasome inhibitor such as bortezomib and steroids), followed by high-dose melphalan therapy (HDT) and ASCT, and finally maintenance therapy with lenalidomide. For patients not eligible for ASCT, there are two or three different drug options, using bortezomib, lenalidomide, melphalan, cyclophosphamide, thalidomide, and steroids. At relapse, therapeutic options may be based on numerous drug combinations of doublets or triplets of several different drugs such as bortezomib, carfilzomib, ixazomib, daratumumab, lenalidomide, pomalidomide, and elotuzumab. The choice of therapy depends on several factors, including prior exposure to a drug class, prior response and duration of response, residual toxicity, patient’s frailty, etc. There is currently no prognostic or predictive factor or staging system to routinely use in order to define a risk-adapted strategy, and more research in this field is needed [77].

**Figure 1:**
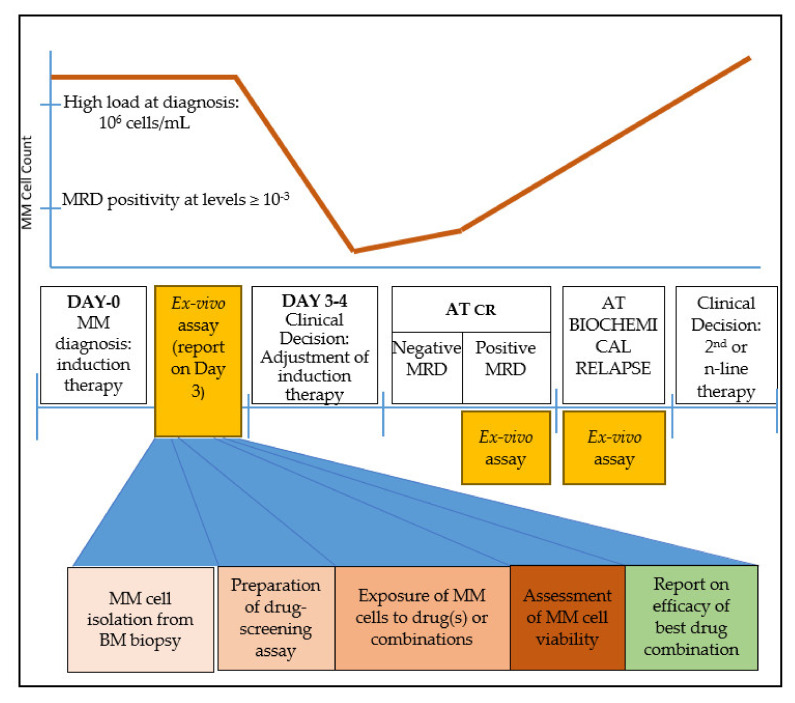
Clinical timeline of multiple myeloma (MM) treatment and suggested timing for ex vivo drug-screening assay. An abundance of MM cells is critical for the successful performance of the assay. Such windows appear immediately after diagnosis, at complete response (CR) when minimal residual disease (MRD) contains sufficient tumor burden, and at biochemical relapse. The steps for a clinically relevant ex vivo screening assay are sample collection and MM cell isolation in adequate numbers; exposure of cells to the drug(s); assessment of cell viability; and finally a report on the most effective drug(s) or drug combinations.

After a holistic examination of the treatment course of MM, we believe that there are three time points where an ex vivo drug-testing platform could be helpful for MM patients to provide clinically-relevant information that could assist oncologists’ decision-making (Figure 1):

(A) immediately after MM diagnosis, to adjust the ideal drug combination for induction therapy. The strength of this time point is the abundance of MM cells that can be isolated and used for drug screening.

(B) at minimal residual disease (MRD) evaluation, if the clone is detected at levels ≥ 10^−3^. At this point, the model may be informative of the presence of a residual resistant clone that may eventually lead to relapse. The disadvantage stands in cases where the MRD myeloma burden is low and therefore an inadequate number of cells would be isolated for drug screening. Moreover, since MRD+ cells would probably be a minority of the total plasma cell population, an FACS-sorting approach would be essential to phenotypically discriminate aberrant plasma cells from their normal plasma cell counterpart.

(C) upon biochemical relapse. This is probably the ideal time-point where a drug assay would provide essential information for drug selection or drug combination. MM cells are abundant, while available clinical information based on previous exposure to various drugs can be used to cross-validate the results. Mathematical models that incorporate earlier response(s) to therapy may also help improve prediction.

## 14. Conclusions—The Way for an Effective Ex Vivo BM Model for MM Patients

After reviewing all relevant models, the question that arises is whether we need to combine various elements in order to establish and clinically validate a feasible drug-screening model simulating the BM microenvironment. Having thoroughly examined research data available to date, we show that each platform demonstrates particular advantages either as an overall approach or in specific aspects of BM simulation.

**Figure 2:**
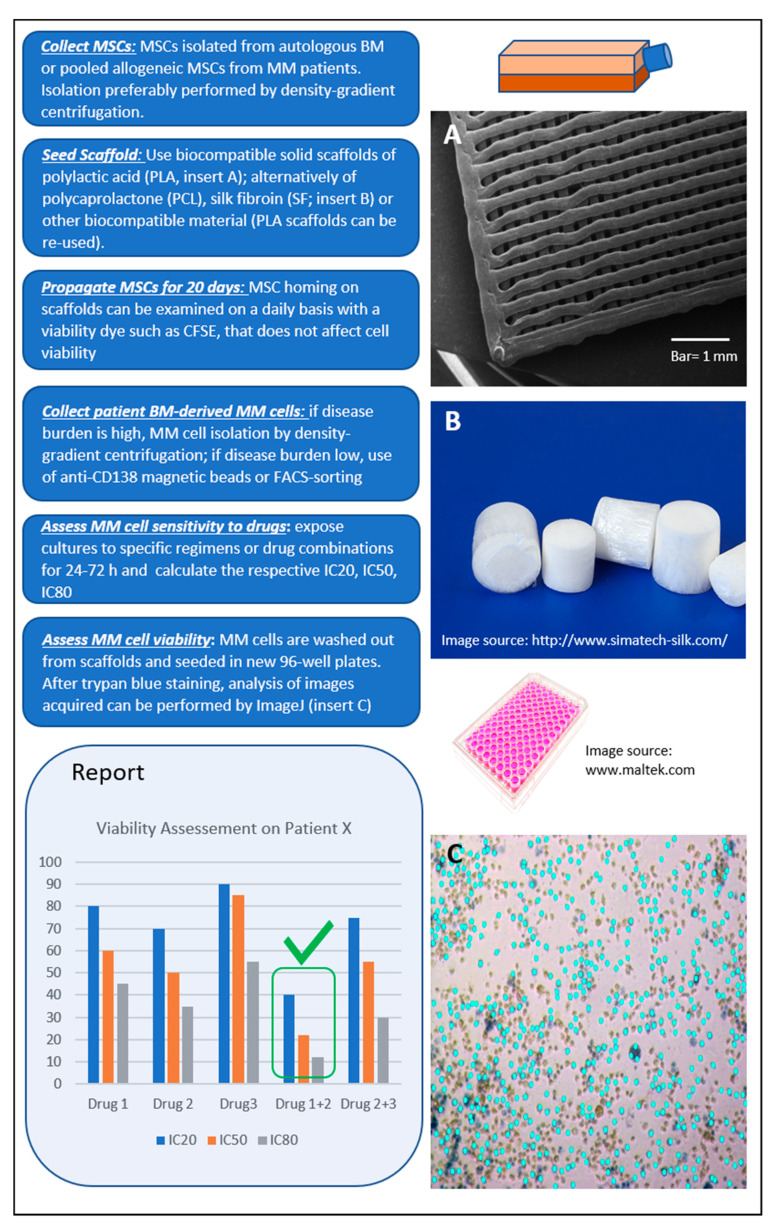
Proposed ex vivo 3D assay simulating the bone marrow (BM) microenvironment of multiple myeloma (MM) patients. Workflow shown from top left to bottom left. The report contains information on patient-specific effectiveness of tested therapeutic agent(s) selected by clinicians. In the example shown, the best response of patient X is to Drugs 1 + 2 (green tick mark).

We propose a model (Figure 2) with the following desired characteristics: (a) low cost, including consumables and equipment required; (b) high-throughput option for parallel screening of many drugs and drug combinations in a single run; and (c) easy workflow. Based on our experience, MSCs can efficiently populate polylactic acid (PLA) scaffolds of ca. 100 nm pore size within 3–4 weeks. The use of biocompatible PLA scaffolds pre-seeded with pooled BM MSCs in a 96-well plate format meets the aforementioned characteristics and has potential for the development of an optimized short-term (1–3 days) ex vivo drug-screening assay. Primary MM cells seeded to the wells are let home to the scaffolds, exposed to drugs or drug-combinations for 24–72 h, and finally are washed out for viability assessment (Supplementary Figure S1; unpublished data).

Recent developments and intense research have brought to light numerous novel regimens for the clinical management of MM; however, a simple, cost-effective, and reliable ex vivo model that applies in the clinical practice, eventually expanding beyond therapy selection to disease state prediction (e.g., discriminating between MGUS and smoldering myeloma or more importantly between high-risk smoldering MM and active myeloma) is still lacking. Translating a preclinical model from bench to bedside requires intensive research, vigorous development, immense validation, and funding. We expect that research in this field will be more intense in the years to come.

## Figures and Tables

**Table 1:** Selected ex vivo 3D models simulating the bone marrow environment of multiple myeloma (MM) patients.

Year	Reference	Type of Model	Drugs Tested	Model Composition	Cell Types Cocultured (BM Cells/MM Cells)	Viability Assessment	Duration of Test	Feasibility	Cost
**2008**	Kirshner et al. [52]	Gel scaffold	MELPH, BTZ	Recombinant bone marrow; culture plates with fibronectin/collagen, ECM mixture of Matrigel/fibronectin	primary MSCs/primary MM cells	Annexin V analysis of CD38+ cells by flow cytometry; treatment efficacy evaluated using RQ-PCR to detect genomic clonotypic IgH VDJ	⏰⏰⏰	★★☆	$$
**2018**	Huang et al. [54]	Gel scaffold	Stattic (a STAT3 inhibitor), BTZ	Fibronectin/Collagen scaffolds in 48-well plates seeded with cells in Matrigel	primary MSCs/U266, RPMI8226	Cell viability measured by CellTiter 96 Assay (i.e., MTT) or trypan blue exclusion assay; apoptosis measured by an Annexin V Apoptosis Detection Kit	⏰	★★★	$$
**2015**	De la Puente et al. [55,56]	Gel Scaffold	DOXO, BTZ, CFZ	3D Tissue-Engineered BM cultures; scaffold formed by cross-linking plasma fibrinogen with CaCl2 and tranexamic acid	human umbilical vein endothelial cells, primary CD138+ and CD138- (stromal) cells/MM1s, H929, RPMI8226, MM1s-GFP-Luc	Cell survival analyzed by flow cytometry against an internal standard	⏰⏰	★☆☆	$$
**2016**	Jakubikova et al. [57]	Gel Scaffold	POM, LEN, THAL, BTZ, CFZ, DOXO, DEX, MELPH	PuraMatrix hydrogel	primary MSCs/primary MM cells, OPM1, KMS11, OCIMY5	CFSE and Annexin V-PE apoptosis assays by flow cytometry	⏰	★★★	$$
**2017**	Arhoma et al. [58]	Gel Scaffold	TRAIL, HDAC inhibitors	3D tumor spheroid cultures on alginate beads	NCI-H929, RPMI 8226, OPM-2, JJN-3, U266/one primary cell culture generated from a plasma cell leukaemia patient (ADC-1)	Caspase-3 activity measured by flow cytometry; cell viability based on CellTiter-Glo luminescent assay	⏰⏰	★★☆	$
**2017**	Ji et al. [59]	Gel Scaffold	BTZ, LEN and THAL combined	Collagen gel; computational model consisting of a hybrid multi-scale agent-based model	primary MSCs, HS-5 MSC cell line/U266RPMI 8226	Cell viability determined by MTT assay	⏰⏰	★☆☆	$
**2018**	Braham et al. [60,61]	Gel Scaffold	TEGs (αβT cells engineered to express a defined γδTCR)	3D co-cultures using Matrigel; MM cell lines and patients’ cells labelled with a Vybrant Multicolor Cell-Labeling Kit (DiO, DiI, DiD) co-cultured with MSCs	primary MSCs, embedded endothelial progenitor cells /OPM2, U266 and L363	Viability assessed with calcein staining by confocal imaging	⏰⏰	★★★	$$$
**2017**	Silva et al. [62]	Gel Scaffold	31 drugs	384 or 1536 multi-well plates with collagen type I and previously established MSCs	primary MSCs/primary MM cells, MM1.S	Digital Image Analysis algorithm computing differences in sequential images and identifying live cells	⏰	★★☆	$
**2014**	Reagan et al. [63]	Solid scaffold	BTZ	Porous, aqueous, silk fibroin scaffolds with pores 500-600 μm cut into cylinders; fibrinogen, thrombin and Matrigel mixed with cells before seeding	primary MSCs (healthy and patient derived)/primary MM cells, MM1S, OPM2	Calcein on a confocal microscope or LIVE/DEAD Fixable Red Dead Cell Stain kit	⏰⏰⏰	★★☆	$$
**2018**	Bellloni et al. [64]	Bioreactor	ΒΤΖ, MELPH, DEX, anti-VLA-4 mAb natalizumab	Dynamic cultures in RCCS Bioreactor	primary MSCs osteoblasts, HS-5 cell line, murine L-fibroblasts, human umbilical vein endothelial cells/primary MM cells, MM1.S, U266, RPMI.8226	Annexin V analysis of CD38+ cells by flow cytometry	n/a	★☆☆	$$$
**2017**	Bonomi et al. [65]	Bioreactor	PTX, BTZ	Dynamic cultures in RCCS Bioreactor	MSCs (Lonza, USA)/CFPAC-1 adenocarcinoma cell line, RPMI8226	MTT assay	⏰⏰	★★☆	$$$
**2013**	Khin et al. [66]	Fluidics	MELPH, BTZ	3D cell culture slides (µ-slide Chemotaxis 3D *Ibitreat* from Ibidi, LLC) with bovine collagen type I	primary MSCs/primary MM cells, RPMI-8226, HS-5, H929, 8226/LR-5	Assessment of cell viability through membrane motion detection with ImageJ	⏰	★★☆	$$
**2015**	Martowicz et al. [67]	Animal	BTZ	Spheroid grafted on chorioallantoic membrane of chicken embryos	primary MSCs/OPM-2, RPMI-8226	Tumor cell mass measurement of eGFP contents with GFP-ELISA	⏰⏰	★☆☆	$
**2011**	Calimeri et al. [68]	Animal	DEX, BTZ	PCLS scaffold cylinders surgically implanted subcutaneously into SCID mouse flank	primary MSCs (human and mouse derived)/OP9	Detection of paraprotein levels in mouse sera; detection of MM cell apoptosis in retrieved PCLSs	⏰⏰⏰	★☆☆	$$$

BTZ, Bortezomib; CFZ, Carfilzomib; DEX, dexamethasone; DOXO, Doxorubicin; HDAC, histone deacetylase; LEN, Lenalidomide; MELPH, Melphalan; POM, Pomalidomide; PTX, Paclitaxel; THAL, Thalidomide; TRAIL, Tumour Necrosis Factor-Related Apoptosis-Inducing Ligand. ⏰, week; ⏰⏰, month; ⏰⏰⏰, over a month; ★★★, feasible under usual laboratory conditions; ★★☆, needs some specific techniques; ★☆☆, difficult implementation; $, estimated cost ≤ 10k euros; $$, cost ≤ 100k euros; $$$, cost ≥ 100k euros. Other abbreviations: BM, bone marrow; CFSC, carboxyfluorescein succinimidyl ester; ECM, extracellular matrix; GFP, green fluorescence protein; MSCs, marrow stromal cells; MTT, 3-(4,5-dimethylthiazol-2-yl)-2,5-diphenyltetrazolium bromide; PCLS, poly-ε-caprolactone polymeric scaffold; PE, phycoerythrin; RCCS, RSS Bioreactor; RQ-PCR, real-time quantitative polymerase chain reaction; SCID, severe combined immunodeficient; TCR, T cell receptor.

## Supplementary Materials

The following are available online at https://www.mdpi.com/2072-6694/12/8/2006/s1, Figure S1: Experimental setup of a 3D BM culture simulation.
