# Supplementary material for: Ex Vivo Models Simulating the Bone Marrow Environment and Predicting Response to Therapy in Multiple Myeloma
Source: Cancers (Basel). 2020 Jul 22;12(8):2006. doi: 10.3390/cancers12082006 (PMC7463609; doi:10.3390/cancers12082006)
Supplement: Supplementary file 1 [file cancers-12-02006-s001.pdf]

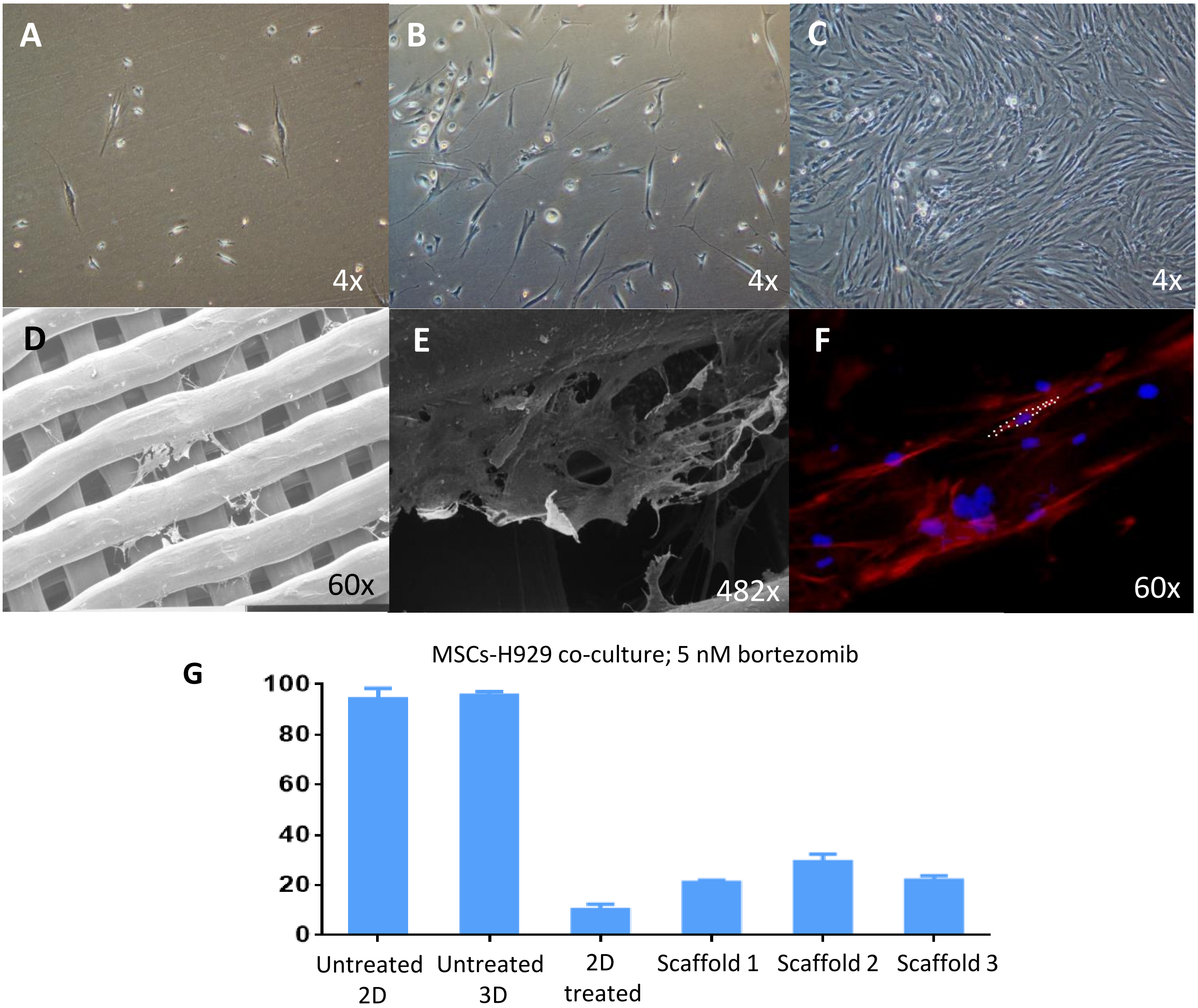

Supplementary Figure 1. Experimental setup of a 3D BM culture simulation. MM patient-derived MSCs in culture at day 0 **(A)**, day 10 **(B)** and day 20 **(C)**. SEM imaging of primary MSCs on PLA scaffolds showing cells growing between grid filaments **(D, E)**. Confocal imaging (phalloidin/red – DAPI/blue) of MSCs on PLA scaffolds **(E)**. Indicative assay results of MSCs-H929 MM cell co-culture using PLA scaffolds of different pore sizes (1, 2 and 3) in a 96-well format **(G)**. MSCs initially grew on the scaffolds, H929 cells were added and exposed to 5 nM bortezomib (IC80). At 24 h, H929 were washed out and stained with Annexin V-propidium iodide for viability assessment. Cell viability was higher in 3D *versus* 2D cultures (*unpublished data*).
